# The Placental Microbiota Is Altered among Subjects with Gestational Diabetes Mellitus: A Pilot Study

**DOI:** 10.3389/fphys.2017.00675

**Published:** 2017-09-06

**Authors:** Jia Zheng, Xinhua Xiao, Qian Zhang, Lili Mao, Miao Yu, Jianping Xu, Tong Wang

**Affiliations:** Department of Endocrinology, Key Laboratory of Endocrinology, Ministry of Health, Peking Union Medical College Hospital, Diabetes Research Center of Chinese Academy of Medical Sciences and Peking Union Medical College Beijing, China

**Keywords:** gestational diabetes mellitus, placenta, microbiota, 16S rRNA gene, clinical characteristics

## Abstract

Gestational diabetes mellitus (GDM) has significant implications for the future health of the mother and child. However, the associations between human placental microbiota and GDM are poorly understood. We aimed to profile the placental microbiota of GDM and further define whether or not certain placental microbiota taxon correlates with specific clinical characteristics. Placenta were collected from GDM women and women with normal pregnancies (*n* = 10, in each group) consecutively recruited at Peking Union Medical College Hospital. The anthropometric parameters of mother and infant, and cord blood hormones, including insulin, leptin and insulin-like growth factor-1 (IGF-1) were measured. Bacterial genomic DNA was isolated using magnetic beads and the human placental microbiota was analyzed using the Illumina MiSeq Sequencing System based on the V3-V4 hypervariable regions of the 16S rRNA gene. It showed there was no statistical difference in the clinical characteristics of mothers and infants, such as BMI at the beginning of pregnancy and gestational weight gain (GWG), birth weight, and cord blood hormones, including insulin, leptin and IGF-1. We found that the placental microbiota is composed of four dominant phyla from Proteobacteria (the most abundant), Bacteroidetes, Actinobacteria and Firmicutes, with the proportion of Proteobacteria increased, and Bacteroidetes and Firmicutes were decreased of women with GDM. Further analyses suggested that bacterial taxonomic composition of placentas from the phylum level down to the bacteria level, differed significantly between women with GDM and non-GDM women with normal pregnancies. Regression analysis showed a cluster of key operational taxonomic units (OTUs), phyla and genera were significantly correlated with GWG during pregnancy of mothers, and cord blood insulin, IGF-1 and leptin concentrations. In conclusion, our novel study showed that a distinct placental microbiota profile is present in GDM, and is associated with clinical characteristics of mothers and infants. This study contributes to the theoretical foundation on the potential relationship between placental microbiota and GDM.

## Introduction

Obesity and type 2 diabetes mellitus (T2DM) is increasing rapidly. A particularly alarming trend is that gestational diabetes mellitus (GDM) has also increased dramatically, along with increasing rates of obesity (Dabelea et al., 2005; Sacks et al., 2012). In 2015, the International Diabetes Federation (IDF) estimated that one in seven births is affected by GDM (International Diabetes Federation, 2015). World Health Organization stated in 2016 that about 50% female population are currently overweight (World Health Organization, 2016) and are at high risk of developing GDM. Women with GDM had considerable risks of developing maternal and fetal morbidity and mortality (Hillier et al., 2007). For the mothers, it can increase the susceptibility of undergoing a Caesarian section, preeclampsia and postpartum development of T2DM (Capula et al., 2014). Adverse outcomes in infants include macrosomia, shoulder dystocia, preterm birth, and metabolic dysfunction such as neonatal hypoglycemia (Hillier et al., 2007; Metzger et al., 2008; Catalano et al., 2012). In addition, offspring of women with GDM may be more likely to develop obesity, insulin resistance and early-onset T2DM in young adulthood (Kubo et al., 2014), which has been clearly revealed by the “Developmental Origins of Health and Disease (DOHaD)” hypothesis (Wallack and Thornburg, 2016; Baird et al., 2017). Therefore, GDM not only increases adverse pregnancy outcomes, but also has long-term deleterious effects on the metabolic health of mothers and their offspring.

The structure and function of human microbiome across multiple sites, including mouth, stomach, gut, urogenital tract, lungs, stool, and skin, has been demonstrated to be linked to several human diseases (Consortium, 2012). A metagenome-wide association study showed that gut microbiota were closely related with T2DM (Qin et al., 2012). However, as an important organ for fetal development, growth and survival during pregnancy, limited studies were about the placenta, especially with the bacteria within it (Watson and Cross, 2005). Until recently, it is widely accepted that the placenta are “sterile,” that is essential to protect the fetus from infections (Wassenaar and Panigrahi, 2014). However, increasing evidence indicate that microbes is present in the normal placenta. In a cohort with 320 subjects, Aagaard et al. found a unique placental microbiome profile, composed of non-pathogenic commensal microbiota and were most akin to the oral microbiome (Aagaard et al., 2014; Pelzer et al., 2016). Compelling evidence suggests that placental microbiota are associated with pregnancy outcomes and human diseases. Doyle et al. showed that preterm labor were correlated with distinct microbial communities in placentas (Doyle et al., 2014). One previous study suggested that excess gestational weight gain (GWG) was correlated with an altered placental microbiome among women who experienced a preterm birth (Antony et al., 2015). Our previous data showed that placental microbiome was distinct in full-term neonates with low birth weight (Zheng et al., 2015). However, the relationship between placental microbiota and GDM are poorly understood. Given that GDM is a high risk factor of multiple adverse outcomes, we aimed to study the placental microbiota in GDM and further define whether or not certain placental microbiota taxon correlates with specific clinical characteristics.

## Materials and methods

### Study subjects and eligibility criteria

This study was approved by the Institutional Review Board and Ethics Committee of Peking Union Medical College Hospital (PUMCH, No: S-003) and the study conformed to the relevant regulatory standards. All pregnant women who come to delivery at Peking Union Medical College Hospital (Beijing, China) were consecutively recruited for our study. Women who had the following criteria were excluded from the study: (1) women who gave birth with a gestational age <37 or >42 weeks, (2) women were diagnosed as diabetes mellitus (World Health Organization, 1999), hypertension, nephropathy, thyroid dysfunction, and any other severe illnesses before pregnancy, (3) women were treated with any medications during pregnancy, (4) women with multiparous pregnancy and fetuses with genetic or congenital malformations, asphyxia at birth, neurological dysfunction or any other diseases, (5) the presence of any antepartum infections and antibiotic treatment during pregnancy. After informed written consent was obtained, 20 pregnant women, including 10 with GDM and 10 non-GDM (NDM) women with normal pregnancies were included in our study. All mothers are from Chinese Han ethnicity and had undergone a 75-g OGTT following the standard protocol at 24–28 weeks of gestation. GDM was diagnosed when the fasting plasma glucose ≥5.1 mmol/L or 1 h post-OGTT glycemia ≥10.0 mmol/L or 2 h post-OGTT glycemia ≥8.5 mmol/L, according to the criteria set by International Association of Diabetes and Pregnancy Study Groups (IADPSG) (Metzger et al., 2010; American Diabetes Association, 2014).

Information on maternal characteristics was extracted from standardized medical records and subjects interviews, including age, body weight (BW), and body mass index (BMI) at the beginning of the first trimester, medical history, diabetes status before and during pregnancy, and GWG. BMI was calculated as body weight (kg) /height (m)^2^. The age and BMI at the beginning of the first trimester were matched between pregnant women with GDM and non-GDM women, in order to avoid any bias related to age and gestational obesity/overweight. All the pregnant women with GDM were intervened by lifestyle changes, including diets and exercise, in order to avoid any bias due to medications. In addition, several studies have shown the mode of delivery (Cesarean section vs. vaginal delivery) could affect the gut microbiota composition of newborns (Mackie et al., 1999; Dominguez-Bello et al., 2010), which may affect placental microbiota. Thus, we only included women in labor who delivered by cesarean section, in order to minimize the potential confounders of delivery mode and placenta microbiota.

### Anthropometric measurements of infants

All infants were born at full-term, with gestational age between 37 and 42 weeks. The anthropometric measurements of infants were collected according to our previous publications (Zheng et al., 2014, 2015). The information was obtained by two trained research nurses and assistants. Measurements include gestational age, sex, birth weight, placenta weight, body length, ponderal index, head circumference, and neonatal complications. Gestational weeks were according to the last menstrual period and were confirmed by ultrasound results. Birth weight and placenta weight, body length, and head circumference were measured immediately after birth, using corresponding measurement tools. Ponderal index was calculated as birth weight (kg) /body length (m)^3^. Table 1 showed the clinical characteristics and anthropometric measurements of mothers and infants.

**Table 1:** Clinical characteristics and biochemical variables.

	**NDM**	**GDM**	***P*-value**
**MOTHERS**			
Maternal age (years)	31.21 ± 4.34	32.09 ± 3.21	0.58
BW 1st (kg)	55.68 ± 7.72	53.14 ± 5.31	0.36
BMI 1st (kg/m^2^)	20.53 ± 2.19	20.40 ± 1.90	0.88
GWG (kg)	15.57 ± 4.38	13.59 ± 4.94	0.30
BW 3rd (kg)	71.25 ± 8.13	66.73 ± 6.64	0.15
BMI 3rd (kg/m2)	26.28 ± 1.92	25.61 ± 2.08	0.41
BMI gain (kg/m2)	5.74 ± 1.55	5.20 ± 1.78	0.42
**INFANTS**			
Sex (Male %)	57%	64%	/
Gestational week (weeks)	39.44 ± 0.98	39.38 ± 0.37	0.84
Birth weight (g)	3287.14 ± 200.13	3246.36 ± 159.33	0.59
Body length (cm)	50.07 ± 1.49	49.64 ± 1.12	0.43
Ponderal index (kg/m3)	26.21 ± 1.56	26.59 ± 1.75	0.58
Head circumference (cm)	34.13 ± 1.07	33.95 ± 0.68	0.63
Placenta weight (g)	639.64 ± 106.69	614.55 ± 78.67	0.52
**CORD BLOOD PARAMETERS**			
Insulin (μU/ml)	13.10 ± 6.24	10.76 ± 3.65	0.41
Leptin (ng/ml)	9.74 ± 7.37	5.45 ± 2.08	0.18
IGF-1 (ng/ml)	45.23 ± 13.73	44.40 ± 14.79	0.91

*Data reported as the mean ± Standard Deviation (S.D.). n = 10, in each group. BW, body weight; BMI, body mass index; BW 1st, body weight at the beginning of 1st trimester; BW 3rd, body weight at the end of 3rd trimester; BMI 1st, BMI at the beginning of 1st trimester; BMI 3rd, body mass index at the end of 3rd trimester; GWG:gestational weight gain; GDM, gestational diabetes mellitus; NDM, Non-GDM; IGF-1, insulin-like growth factor-1*.

### Cord blood hormones analysis

Cord blood were collected from the umbilical cord vein immediately after delivery with ethylene diamine tetraacetic acid (EDTA) as anticoagulant. All plasma were obtained by centrifugation at 4,000 × g for 10 min at room temperature and stored in multiple aliquots at −80°C until assays. We measured insulin, leptin and insulin-like growth factor-1 (IGF-1) using the following commercial assays: insulin (radioimmunoassay, HI-14K, Linco Research Inc., St. Charles, Missouri, USA), leptin (radioimmunoassay, HL-81HK, Linco Research Inc., St. Charles, Missouri, USA), and IGF-1 (radioimmunoassay, A15729, Beckman Coulter Diagnostics, Inc., West Sacramento, California, USA), according to the protocol in our previous study (Zheng et al., 2014). The inter-assay and intra-assay coefficients of variation for each of these assays were all <10%. All samples were tested in duplicate and in a blinded manner. The cord blood parameters were shown in Table 1.

### Placental samples collection

All samples were collected by trained pathology personnel, according to previously described protocol (Aagaard et al., 2014; Zheng et al., 2015). More precisely, placentas were placed in sterile containers on ice immediately after Cesarean section delivery in operating room. Then, placentas were weighed and a total of four 1 × 1 × 1 cm placental biopsies were obtained from each placenta, circumferentially excised from separate locations (about 3 cm from the insertion site of umbilical cord). The placental surfaces, including maternal decidua and fetal chorio-namnion were excised and discarded, with only the inner part of the placenta retained, in order to avoid potential contamination during Cesarean section. Personnel collecting samples wore facial masks and sterile gloves to ensure sterility during the whole process. Each placental sample was placed in a sterile, labeled cryovial, then flash-frozen in liquid nitrogen, and stored at −80°C for DNA extraction and further microbiota analysis.

### DNA extraction, PCR amplification and sequencing

DNA was isolated from placental biopsies using the E.Z.N.A.® DNA Kit (Omega Bio-tek, Norcross, GA, USA) in a sterile class II laminar flow hood, following the standard protocol. Extracted DNA was stored at −20°C until further analysis. For each sample, the bacterial 16S ribosomal RNA (rRNA) gene was amplified using bar-coded universal primers targeting the V3-V4 region, listed as: 338F 5′-ACTCCTACGGGAGGCAGCA-3′ and 806R 5′-GGACTACHVGGGTWTCTAAT-3′, and the barcode is a sequence with eight-nucleotide unique to each sample. PCR reactions were carried out in triplicate with the following recipe: 2 μl dNTPs (2.5 mM), 4 μl 5 × FastPfu Buffer, 0.4 μl FastPfu Polymerase, 0.8 μl each forward and reverse primer (5 μM), 10 ng template DNA and PCR-grade water in a final volume of 20 μl. Reactions were performed on a GeneAmp® PCR System 9700 (Thermo Fisher Scientific Inc., Waltham, MA, USA) with conditions: 95°C for 3 min followed by 25 cycles of 95°C for 30 s, 57°C for 30 s, and 72°C for 45 s, with an extension of 10 min at 72°C. Samples that contained known 16S rRNA gene sequences and those that contained no template were used as positive and negative controls in the PCR reactions, respectively. Replicate amplicons were pooled and bead purified using the AxyPrep DNA Gel Extraction Kit (Axygen Biosciences, Union City, CA, USA). Reaction products were paired-end sequenced using the Illumina MiSeq technology (Illumina Inc., San Diego, CA, USA), with the manufacturer's instruction (Caporaso et al., 2012).

### 16S rRNA gene sequence analysis

The Quantitative Insights Into Microbial Ecology (QIIME) software package was utilized to analyze the 16S rRNA gene raw reads (Caporaso et al., 2010). Reads were discarded from the analysis if the truncated reads that were shorter than 50 bp, if the sequences that overlap were shorter than 10 bp, if reads contained ambiguous characters, if more than 2 nucleotide mismatches in primer matching or if reads could not be assembled. Operational taxonomic units (OTUs) were obtained by clustering the reads with 97% similarity by UPARSE (version 7.1, http://drive5.com/uparse/). Rarefaction analysis and Shannon diversity index were calculated according to the OTUs with their relative abundance by QIIME (Caporaso et al., 2010). The phylogenetic affiliation of each sequence was analyzed by RDP Classifier (http://rdp.cme.msu.edu/) with confidence threshold of 70%, based on the silva (SSU115) 16S rRNA database (Amato et al., 2013). Principal coordinate analysis (PCoA) was calculated with the distance matrix according to the weighted UniFrac algorithm (Lozupone et al., 2006). The differences of bacterial taxa were elucidated by linear discriminant analysis (LDA) effect size (LEfSe). The Huttenhower Galaxy web application (The Huttenhower Lab, Boston, MA, USA, http://huttenhower.sph.harvard.edu/lefse/) was used to draw the cladogram by the LEfSe algorithm (Segata et al., 2011).

### Statistical analysis

Data were represented as mean ± standard deviation (S.D.). The statistical analysis was performed by SPSS 21.0 (SPSS Inc., Chicago, IL, USA). Non-parametric variables were mathematically transformed to improve symmetry. Unpaired *t*-test was used to study differences in continuous variables between groups, such as the clinical characteristics and biochemical variables of mothers and infants. Mann–Whitney *U* test was performed at various taxonomy levels to examine differences in bacterial composition between GDM and NDM groups, such as the relative abundance of phylum and genus. The correlation between microbial composition and individual clinical characteristics was identified using Spearman's analysis. *P* < 0.05 was considered to be statistically significant.

## Results

### Anthropometric measurements and cord blood parameters

The clinical characteristics of mothers, infants and cord blood parameters were shown in Table 1. For the mothers, there is no difference in maternal age between women with GDM and women with normal pregnancies. No significant differences in BW and BMI at the end of third trimester, and GWG during pregnancy were observed between pregnant women with GDM and women with normal pregnancies (Table 1). For the variables of infants, there were no marked differences in anthropometric measurements between children of mothers with GDM and mothers with normal pregnancies, including sex, gestational week, placenta weight, birth weight, ponderal index, body length, and head circumference. Moreover, cord blood insulin, leptin and IGF-1 concentrations were not different between GMD and non-GMD groups (Table 1).

### Characteristics of 16S rRNA gene sequencing

To profile placental microbiota structure differences between GDM and women with normal pregnancies, bacterial 16S rRNA gene V3-V4 region were sequenced by Illumina MiSeq platform. Totally, 509,320 high-quality sequences were gained and the average number of each sample was 25,466. The Good's coverage of each sample was more than 97%, which indicates that the sequences identified can represent the majority of bacteria in each sample. We further compared duplicate samples to test the reproducibility of sequencing technique. It showed that the correlation between duplicate samples was >99.5% at any taxonomy level, indicating that the accuracy and reproducibility of sequencing was reliable for further analysis.

The operational taxonomic unit (OTU) count (159.50 ± 34.57 vs. 176.25 ± 27.86) in GDM placenta was similar to that in control group. The estimators of community diversity (Shannon) and richness (Chao) and were detected and no statistically significant difference of Chao and Shannon indices were observed, demonstrating the community richness and diversity of placental microbiota were parallel between GDM and control groups. Detailed information of these characteristics is shown in Table S1.

### Overall microbial structures of placental microbiota

**Figure 1:**
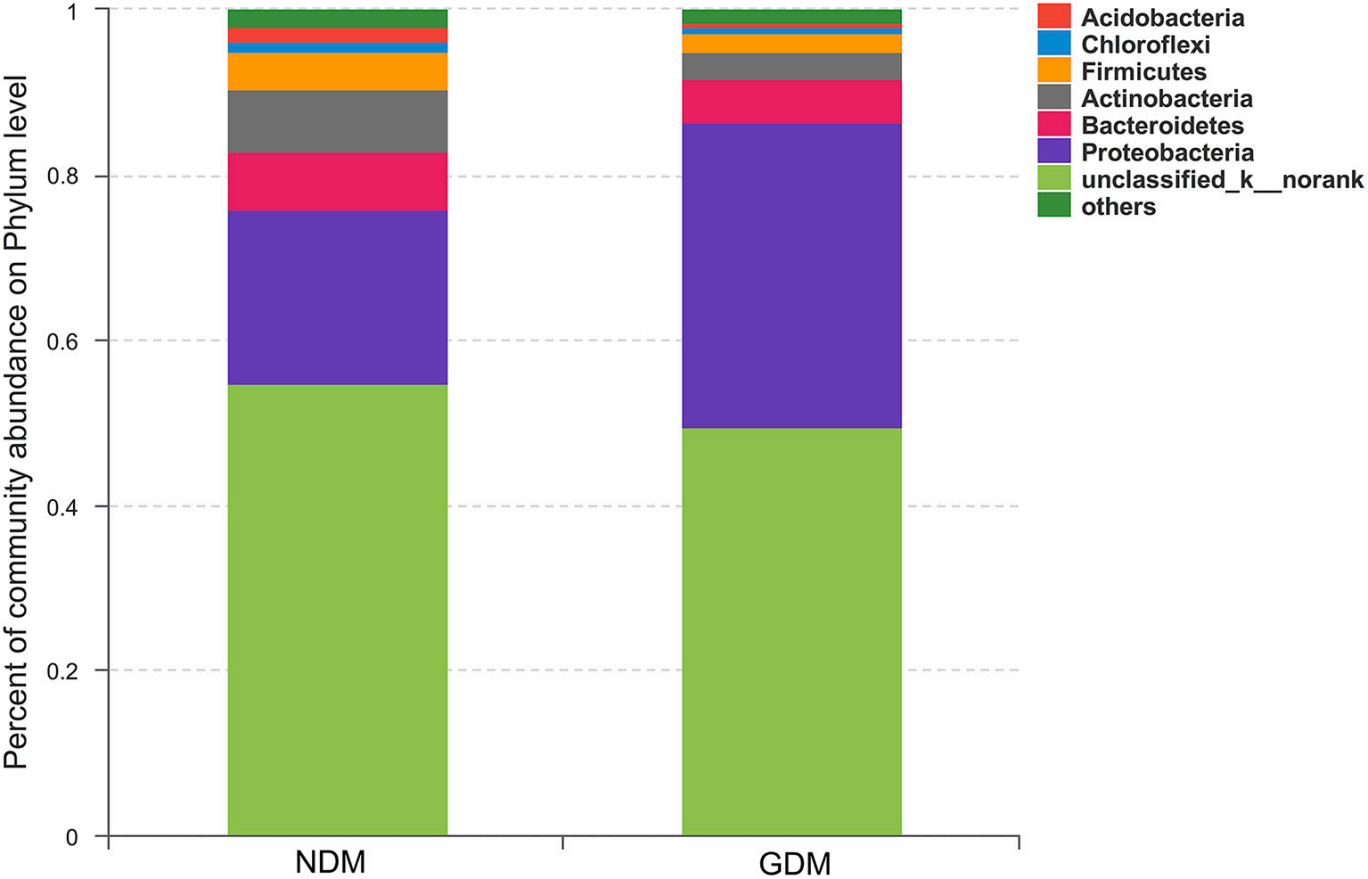
Overall structures of placental microbiota at the phylum level. *n* = 10, in each group. GDM, gestational diabetes mellitus; NDM, Non-GDM.

**Figure 2:**
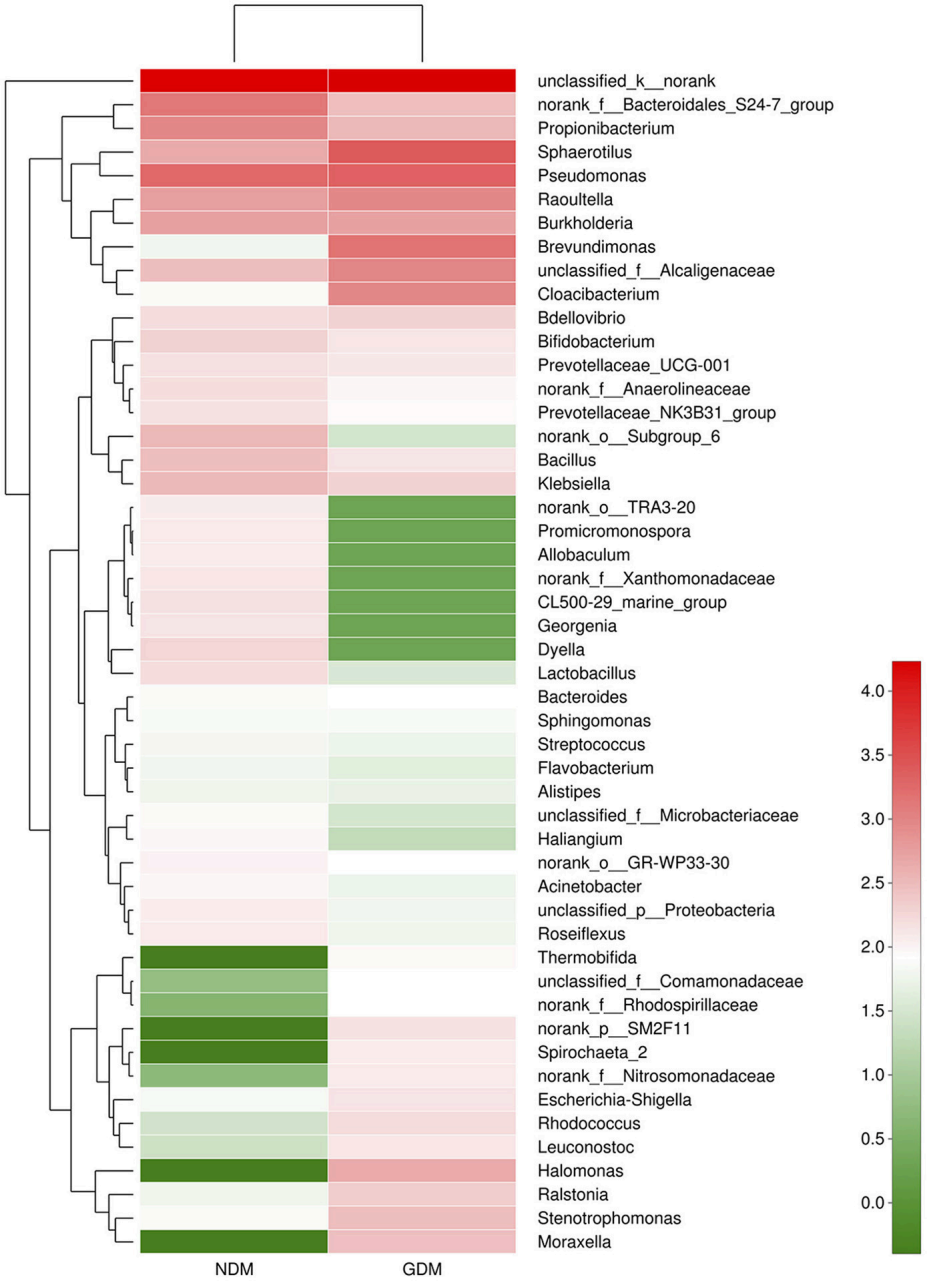
Heatmap analyses of placental microbiota at the genus level. The y axis is a neighbor-joining phylogenetic tree, each row is a different phylotype. The color of the spots in the right panel represents the mean relative abundance of the genera in each group. *n* = 10, in each group. GDM, gestational diabetes mellitus; NDM, Non-GDM.

We studied placental microbiota of women with GDM and non-GDM women with normal pregnancies. Figure 1 shows the overall microbiota structure at the phylum level in each group. The main phyla of GDM and non-GDM groups were Proteobacteria, Bacteroidetes, Actinobacteria, and Firmicutes, with Proteobacteria the most abundant. The proportion of Proteobacteria (36.91 vs. 21.09%) was increased, and the proportion of Bacteroidetes (5.43 vs. 7.03%), Actinobacteria (3.15 vs. 7.35%), and Firmicutes (2.34 vs. 4.53%) were decreased in GDM group, compared to controls. Detailed information of the proportions is shown in Figure S1. The heatmap also demonstrated that microbial structures at genus level of placentas differed significantly between GDM and non-GDM women with normal pregnancies (Figure 2).

**Figure 3:**
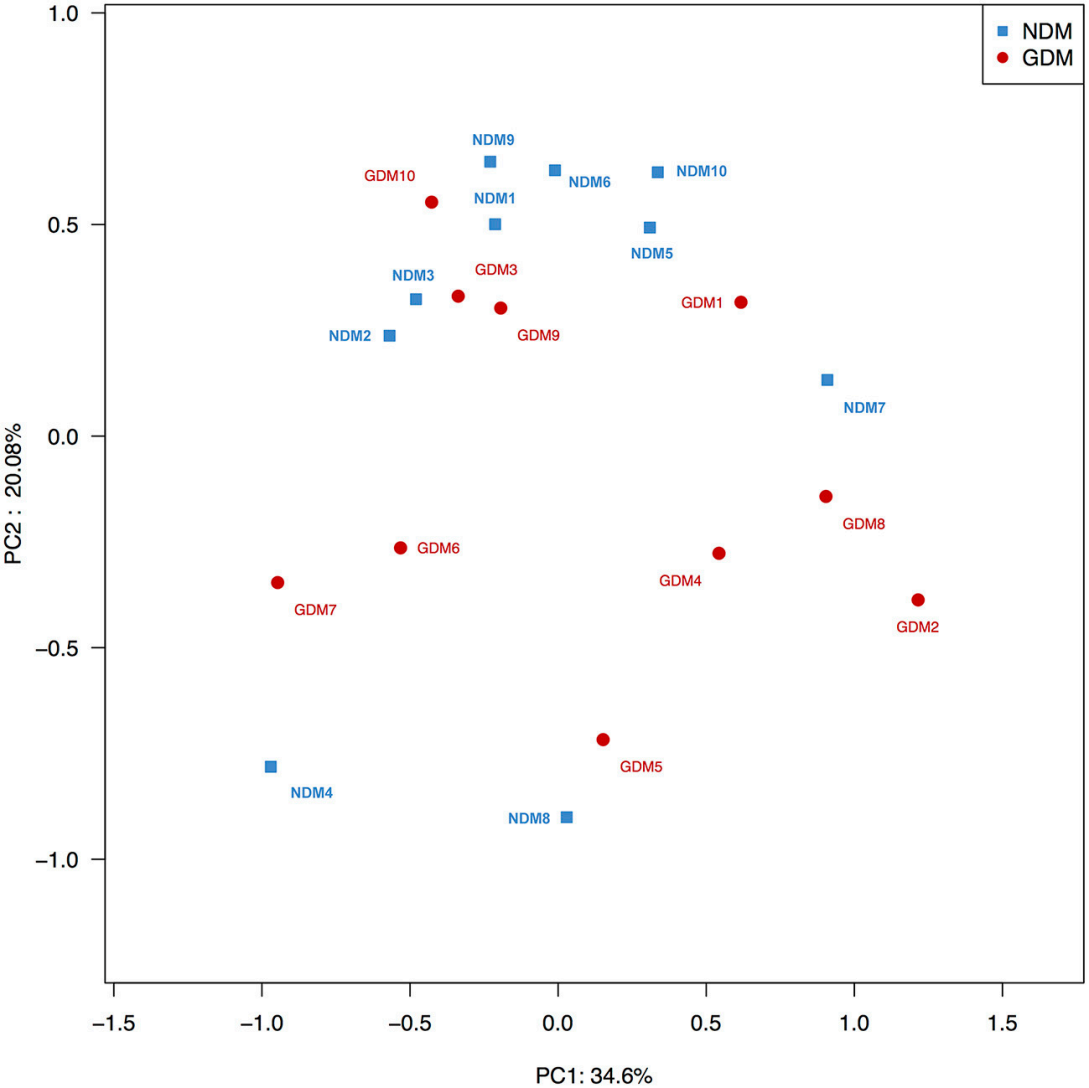
Principal coordinate analysis plots in placental microbiota. Weighted UniFrac PCoA plot based on OTU abundance. Each point represents the placenta microbiota of a newborn, with macrosomia group (red triangle) and control group (green circle). *n* = 10 in each group. GDM, gestational diabetes mellitus; NDM, Non-GDM.

To compare overall placental microbiota structure in pregnant women with GDM and non-GDM women, PCoA according to OTUs of each sample were implemented to provide a glimpse of placental microbial dynamics between GDM and non-GDM groups. The results of PCoA exhibited a significant difference in bacterial structure in placentas between GDM and control groups, with PC1 = 36.6% and PC2 = 20.08% of total variations. It indicated that there was a significant clustering based on glucose metabolic status during pregnancy (Figure 3).

### Taxonomy-based comparisons of placental microbiota between GDM and NDM groups

**Figure 4:**
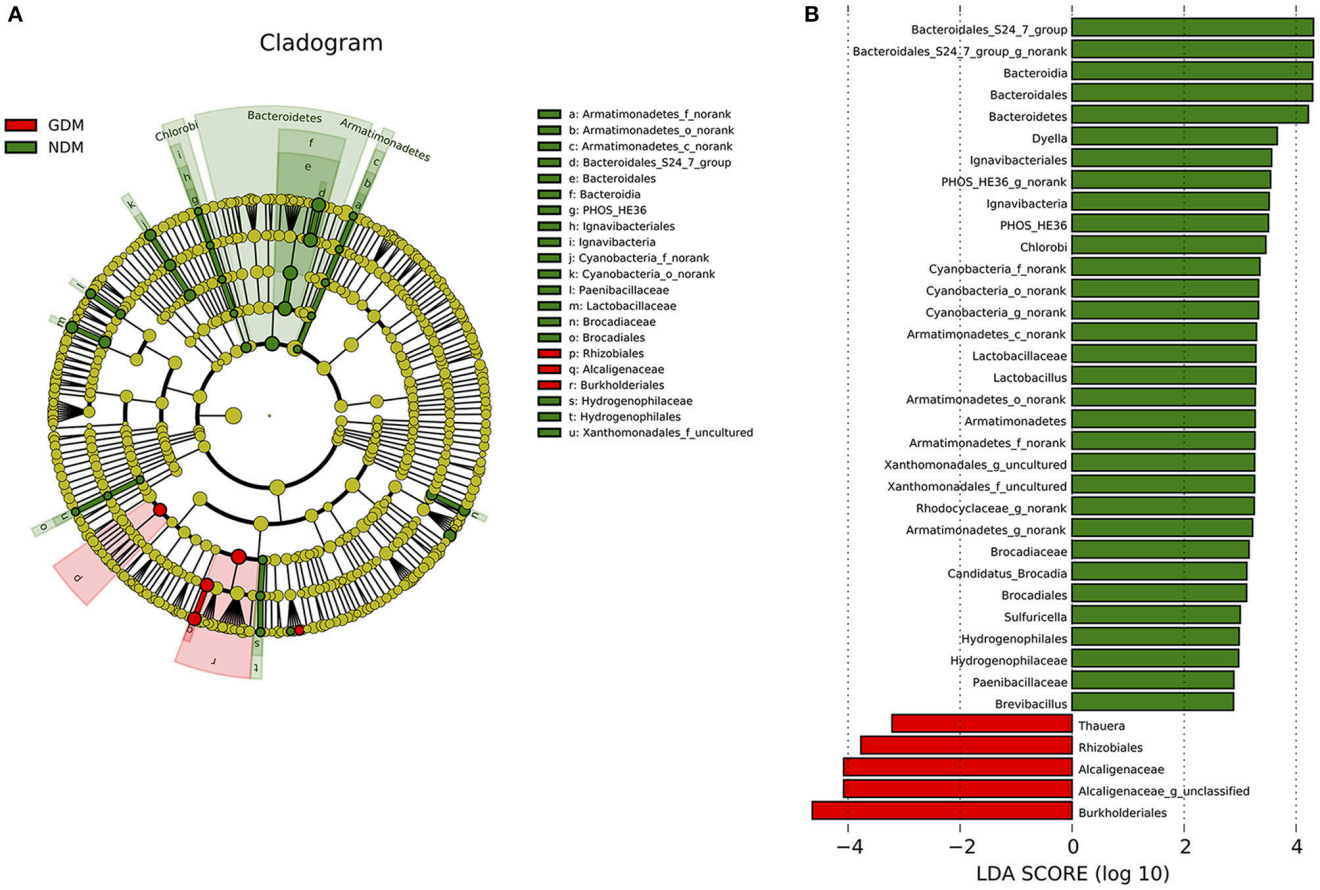
Different structures of placental microbiota between GDM and NDM groups. **(A)** Cladogram representation of different placental microbiota taxa, from the phylum level down to the bacteria level. Red indicates taxa enriched in GDM case subjects, and green indicates taxa enriched in control subjects. The diameter of each circle is proportional to the taxon's abundance. **(B)** Histogram of the LDA scores for differentially abundant taxa (red: GDM; green: NDM). LDA scores were calculated by LDA effect size, using the linear discriminant analysis. *n* = 10, in each group. LDA, linear discriminant analysis; GDM, gestational diabetes mellitus; NDM, Non-GDM.

Significant variations in the composition of placental microbiota were observed from the phylum level down to the bacteria level. A cladogram representation of the significant structure of the placental microbiota is shown in Figure 4A. It includes a list of the predominant bacteria that were significantly changed in either GDM or non-GDM group as determined by LEfSe. The prominent differences between the two groups are shown in Figure 4B.

Illumina MiSeq sequencing data demonstrated that there were statistically significant microbiota compositions in placenta tissue between the two groups at the phylum level. The two dominant phyla, with Proteobacteria (36.91 vs. 21.09%, *P* = 0.046) and Bacteroidetes (5.43 vs. 7.03%, *P* = 0.029) both exhibited statistically significant differences between GDM and NDM placenta tissue. The proportion of Firmicutes (2.34 vs. 4.53%, *P* = 0.083) had a tendency to be decreased in GDM group, compared to controls. The microbial composition was also comparable at the genus level, with 15 genera between GDM and NDM placenta tissues. Bacteroidales_S24-7_group_norank, Lactobacillus, Rhodocyclaceae_norank, Brevibacillus, Armatimonadetes_norank, Candidatus_Brocadia, PHOS-HE36_norank, Sulfuricella, Xanthomonadales_uncultured, Geothrix, TRA3-20_norank, Cyanobacteria_norank and Dyella exhibited a relatively lower abundance in GDM placentas. Alcaligenaceae_unclassified and Thauera were relatively more abundant in GDM group, compared to NDM group (Table 2). Additional information regarding the differences at the taxonomical level of order between GDM and NDM groups can be found in Table S2. Collectively, these observations suggest that microbial composition of placentas differed significantly between GDM and non-GDM women, according to the relative abundance of sequences.

**Table 2:** Phylotypes in placental microbiota significantly different between GDM and NDM groups.

**Taxonomic Rank**	**Specific Taxon**	**NDM (%)**	**GDM (%)**	***P*-value**
phylum	Proteobacteria	21.09	36.91	0.046^*^
phylum	Bacteroidetes	7.03	5.43	0.029^*^
phylum	Firmicutes	4.53	2.34	0.083
phylum	Chlorobi	0.08	0.00	0.006^**^
phylum	Armatimonadetes	0.01	0.00	0.017^*^
phylum	Chloroflexi	1.56	0.71	0.090
genus	Bacteroidales_S24-7_group_norank	4.52	0.97	0.005^**^
genus	Rhodocyclaceae_norank	0.01	0.00	0.006^**^
genus	Brevibacillus	0.04	0.00	0.017^*^
genus	Armatimonadetes_norank	0.01	0.00	0.017^*^
genus	Candidatus_Brocadia	0.01	0.00	0.017^*^
genus	PHOS-HE36_norank	0.00	0.00	0.017^*^
genus	Sulfuricella	0.02	0.00	0.017^*^
genus	Xanthomonadales_uncultured	0.28	0.00	0.017^*^
genus	Lactobacillus	0.54	0.10	0.018^*^
genus	Alcaligenaceae_unclassified	0.98	3.29	0.019^*^
genus	Thauera	0.00	0.05	0.028^*^
genus	Geothrix	0.03	0.00	0.047^*^
genus	TRA3-20_norank	0.36	0.00	0.047^*^
genus	Cyanobacteria_norank	0.27	0.00	0.049^*^
genus	Dyella	0.63	0.00	0.049^*^

*Data of GDM and control groups were showed as relative abundance (%) of phylum and genus in each group. Statistical analysis was performed by the Mann-Whitney U-test. n = 10, in each group*.

* *P < 0.05*,

** *P < 0.001 GDM vs. NDM group. GDM, gestational diabetes mellitus; NDM, Non-GDM*.

### Correlation between placenta microbiota composition and clinical characteristics

**Figure 5:**
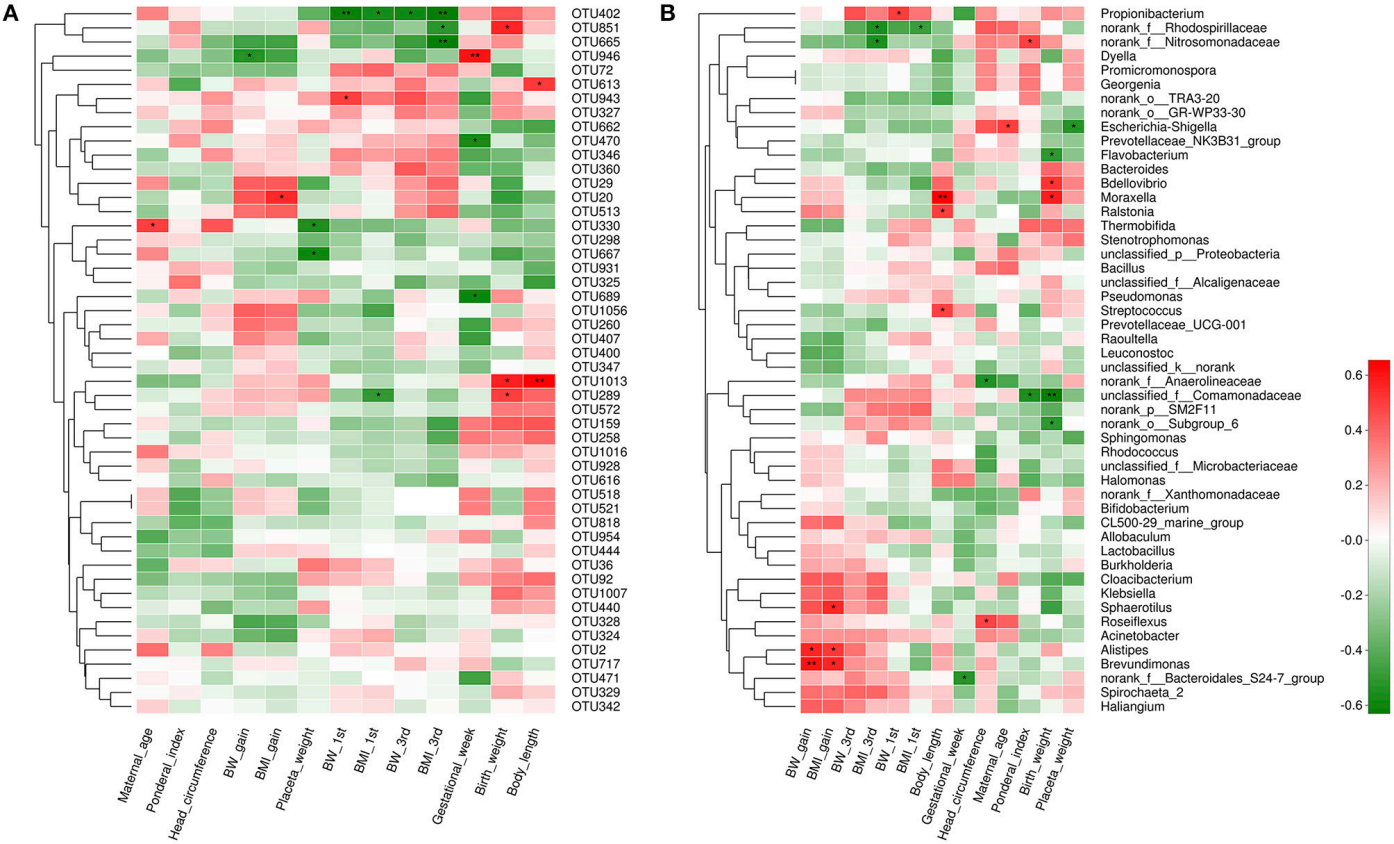
Correlation analyses between placental microbiota and clinical characteristics at the OTU and genus level. **(A)** OTUs were significantly correlated with clinical characteristics. **(B)** Correlation analysis between identified genera and clinical characteristics. Correlation analyses between relative abundance (%) of specific OTU and genus, and clinical parameters were performed by using Spearman's correlation analyses. *n* = 10, in each group. Asterisks represents the specific OTU and genus whose abundance were significantly correlated with certain clinical parameters. The color of the spots in the right panel represents *R*-value of Spearman's correlation between the OTU and genus and clinical parameters. OTU, operational taxonomic unit; BW, body weight; BMI, body mass index; BW 1st, body weight at the beginning of 1st trimester; BW 3rd, body weight at the end of 3rd trimester; BMI 1st, BMI at the beginning of 1st trimester; BMI 3rd, body mass index at the end of 3rd trimester.

Next, we aimed to identify specific placenta microbiota compositions that were potentially correlated with certain clinical characteristics. Spearman's correlation analysis was performed between the top 50 OTUs (based on the rank of relative abundance) and specific clinical characteristics of mothers and infants. In total, 13 OTUs were significantly associated with more than one clinical parameter. OTU946 was negatively correlated with GWG, while OTU20 was positively correlated with BMI gain during pregnancy. One critical OTU (OTU402) was simultaneously negatively associated with BW and BMI at the beginning of the first trimester and at the end of the third trimester. Two OTUs were correlated with lower placenta weight. Three OTUs and two OTUs were correlated with elevated birth weight and higher body length, respectively. No OTUs were correlated with ponderal index and head circumferences (Figure 5A).

At the genus level, 17 genera were significantly correlated with at least one clinical parameter. Of note, Brevundimonas, as a genus of Proteobacteria, and Alistipes, as a genus in the phylum Bacteroidetes were both positively correlated with GWG and BMI gain during pregnancy of mothers. The unclassified_f_Comamonadaceae (as a genus of Betaproteobacteria) was negatively correlated with ponderal index and birth weight of infants. More information about the correlation between genera and clinical parameters is shown in Figure 5B. At the phylum level, no statistically significant correlations were observed between anthropometric measurements and the dominant phyla of the placenta microbiota compositions, including Proteobacteria, Bacteroidetes, Actinobacteria, and Firmicutes. However, we found Spirochaetae phylum was positively correlated with BW at the end of the third trimester, overall GWG and BMI gain during pregnancy of mothers. Saccharibacteria was associated with higher ponderal index of infants (Figure S2).

**Figure 6:**
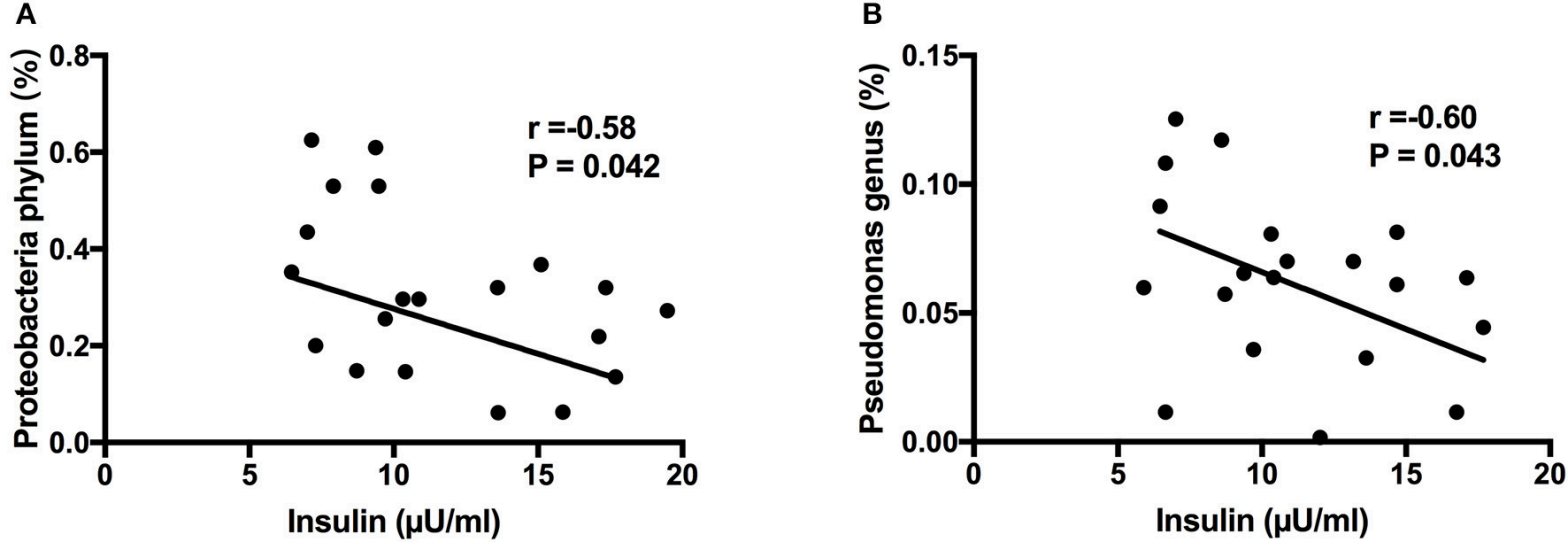
Correlation analyses between placental microbiota and cord blood parameters. **(A)** Proteobacteria phylum was negatively correlated with insulin level. **(B)** Pseudomonas genus was associated with decreased insulin concentration. Correlation analyses were performed by using Spearman's correlation analyses. *n* = 10, in each group. IGF-1, insulin-like growth factor-1.

Next, we aimed to investigate whether specific placenta bacteria were associated with cord blood biochemical parameters, including insulin, leptin and IGF-1. It showed that Proteobacteria, the most abundant phylum of placental microbiota, was negatively correlated with insulin level (*r* = −0.58, *P* = 0.042) (Figure 6A). In addition, Pseudomonas, one dominant genus of placenta bacteria composition, was associated with decreased insulin concentration (*r* = −0.60, *P* = 0.043) (Figure 6B). We also found Staphylococcus (*P* < 0.05) and Allobaculum (*P* < 0.05) genera were both correlated with higher leptin level. Bifidobacterium (*P* < 0.001) and Methylobacterium (*P* < 0.05) genera were both positively associated with IGF-1 level (Figure S3). Thus, these demonstrated that the specific phylum, genus and OTU were significantly correlated with clinical characteristics of the mother and infant, including cord blood insulin, IGF-1 and leptin levels, and may play a critical role in regulating glucose metabolism status, development and growth of the fetus.

## Discussion

GDM has significant implications for the future health of the mother and child. Mothers with history of GDM are more likely to develop postpartum insulin resistance, dyslipidemia, and hypertension (Gobl et al., 2014), placing affected women at risk of T2DM and cardiovascular diseases later in life (Goueslard et al., 2016; Retnakaran and Shah, 2017). The affected offspring are also closely related with risks for metabolic diseases in adult life (Damm et al., 2016). Placental microbiota plays an important role in normal healthy pregnancy, preterm birth, chorioamnionitis and preeclampsia (Pelzer et al., 2016). However, the studies about placental microbiota exposed to GDM are limited. To date, there is only one published study investigating the effect of GDM on the placental microbiota, with decreased abundance of the phylum Bacteroidetes (Bassols et al., 2016). However, our study is more convincing because the delivery mode of all the subjects is consistent. Moreover, our study for the first time examined placenta micorbiota with GDM a Chinese population.

In this study, we investigated placental microbiota profile of GDM and assess the relationship between bacterial taxonomic composition and clinical characteristics of mother and infant. In accordance with previous studies (Aagaard et al., 2014; Antony et al., 2015; Bassols et al., 2016), our study demonstrated that the placental microbiota is composed of four dominant phyla from Proteobacteria (the most abundant), Bacteroidetes, Actinobacteria and Firmicutes, with the proportion of Proteobacteria increased, and Bacteroidetes and Firmicutes were decreased of women with GDM. Further analyses suggested that bacterial taxonomic composition of placentas differed significantly between women with GDM and non-GDM women with normal pregnancies. Additionally, specific metabolic hormones were shown to be associated with bacterial abundance in overweight and obese pregnant women, indicating a connection between metabolic hormones and the microbiota during pregnancy (Gomez-Arango et al., 2016). We found certain specific phylum, genus and OTU were associated with clinical characteristics of mother and infant, including cord blood insulin, IGF-1 and leptin level. It suggests that placenta microbiota may potentially regulate glucose metabolism status, development and growth of the fetus. However, no causal relationship can be inferred from this study due to the cross-sectional design, and more evidence and further investigation are needed.

The origin of the bacteria in the placental microbiota in healthy and unhealthy pregnancies is not fully explored. Three main routes have been proposed: the oral-fetoplacental, gastrointestinal-feto-placental and the genitourinary-fetoplacental routes of placental innoculation (Kuperman and Koren, 2016). Furthermore, the placenta can mediate the negative effects associated with maternal insulin resistance on the fetus. Several studies suggest placentas from humans with GDM and placentas from non-human primates and rodent mothers with insulin resistance have increased hypoxia, oxidative stress, cytokine production, and inflammation in placentas (Radaelli et al., 2003; Li et al., 2013). Previously, it reported a relationship between commensal microbiota and inflammatory parameters (Hanski et al., 2012). Round JL et al. noted that placental microbiota may play a potential role in healthy barrier function and fetal homeostasis (Round and Mazmanian, 2009). It indicated that abundance of Acinetobacter is associated with blood eosinophil counts and expression of anti-inflammatory genes in placenta, such as interleukin-10 (Bassols et al., 2016). Maternal probiotic supplementation with Bifidobacterium lactis, a genus positively correlated with cord blood IGF-1 level in our study, regulated Toll-like receptor (TLR)-related genes expression in the placenta tissue and in the fetal gut (Rautava et al., 2012). Collectively, all the evidence supports the possibility that the changes in placental microbiota in GDM may be related to the increased inflammatory status and activated immune system in women with GMD.

Several strengths and potential limitations should be taken into consideration. Our study has some notable strengths. First, 16S rRNA gene was sequenced by Illumina MiSeq technology, a reliable and widely used high-throughput sequencing platform, which can ensure placental bacteria be successfully identified. Second, the process of sample collection is rigorously sterile and the microbiota analysis is robust, with high quality, accuracy and reproducibility of sequencing. Third, only the fetuses delivered by Cesarean section were enrolled in our study, which can avoid confounding factors with delivery modes. However, several limitations should be taken into consideration. First, the sample size was small. Because the process of subjects' enrollment is strict and complex, we only included babies who were delivered by Cesarean section and mothers whose BMI at the beginning of first trimester were matched. Thus, all these factors restricted the size of our sample. Our ongoing study is aimed at enlarging the sample size. In addition, due to that all the participants included in this study were all recruited in the same hospital, hence potential regional differences in placental microbiota could not be assessed. Our further work will design a multi-center clinical study to fully investigate and compare the placental microbiota among subjects with GDM among different regions. Furthermore, due to the very low sample size, it does not allow to exclude a β error when a significant result is not reached. This, at least in part, could explain the difference with the previous study (Bassols et al., 2016). Second, our study only focused on bacterial taxonomic composition of these communities in placentas rather than on the functional features they express. Thus, the role of intricate microbial communities on maternal and fetal health is still not clear. Therefore, metagenomic analysis in placentas, and even its associations with metabonomics will be determined in our future study. Third, it is a cross-sectional study, which does not allow to infer the causality between placental microbiota and the development of GDM. Thus, well-controlled longitudinal studies are critically important to understanding how changes in the placenta microbiota determine the development of GDM and its effects on mothers and infants. However, our present study is still novel because it provided an important basis for further studies about placenta microbiota of women with GDM.

## Conclusion

In conclusion, our study is novel in showing that a distinct placental microbiota profile is present in GDM, and is correlated with clinical characteristics of mothers and infants, including cord blood biochemical parameters. It contributes to the current theoretical foundation on the potential relationship between placental microbiota and GDM. As the prevalence of GDM has increased over the years, a better understanding of the connections between placental microbiota and the development of GDM would be of great benefit, and help to ensure a healthier future for the mothers and infants. Thus, high-quality, large-scale clinical studies and further pre-clinical experiments to clarify the underlying mechanisms are urgently warranted.

## Author contributions

XX conceived and designed experiments. JZ and LM recruited subjects, collected clinical data and performed placental microbiota analysis. QZ and TW performed cladogram analysis and generated figures. MY and JX analyzed data and reviewed the manuscript. All the authors were involved in writing the paper and had final approval of the submitted and published versions.

### Conflict of interest statement

The authors declare that the research was conducted in the absence of any commercial or financial relationships that could be construed as a potential conflict of interest.
