# Supplementary figures and images for: The Placental Microbiota Is Altered among Subjects with Gestational Diabetes Mellitus: A Pilot Study
Source: Front Physiol. 2017 Sep 6;8:675. doi: 10.3389/fphys.2017.00675 (PMC5592210; doi:10.3389/fphys.2017.00675)

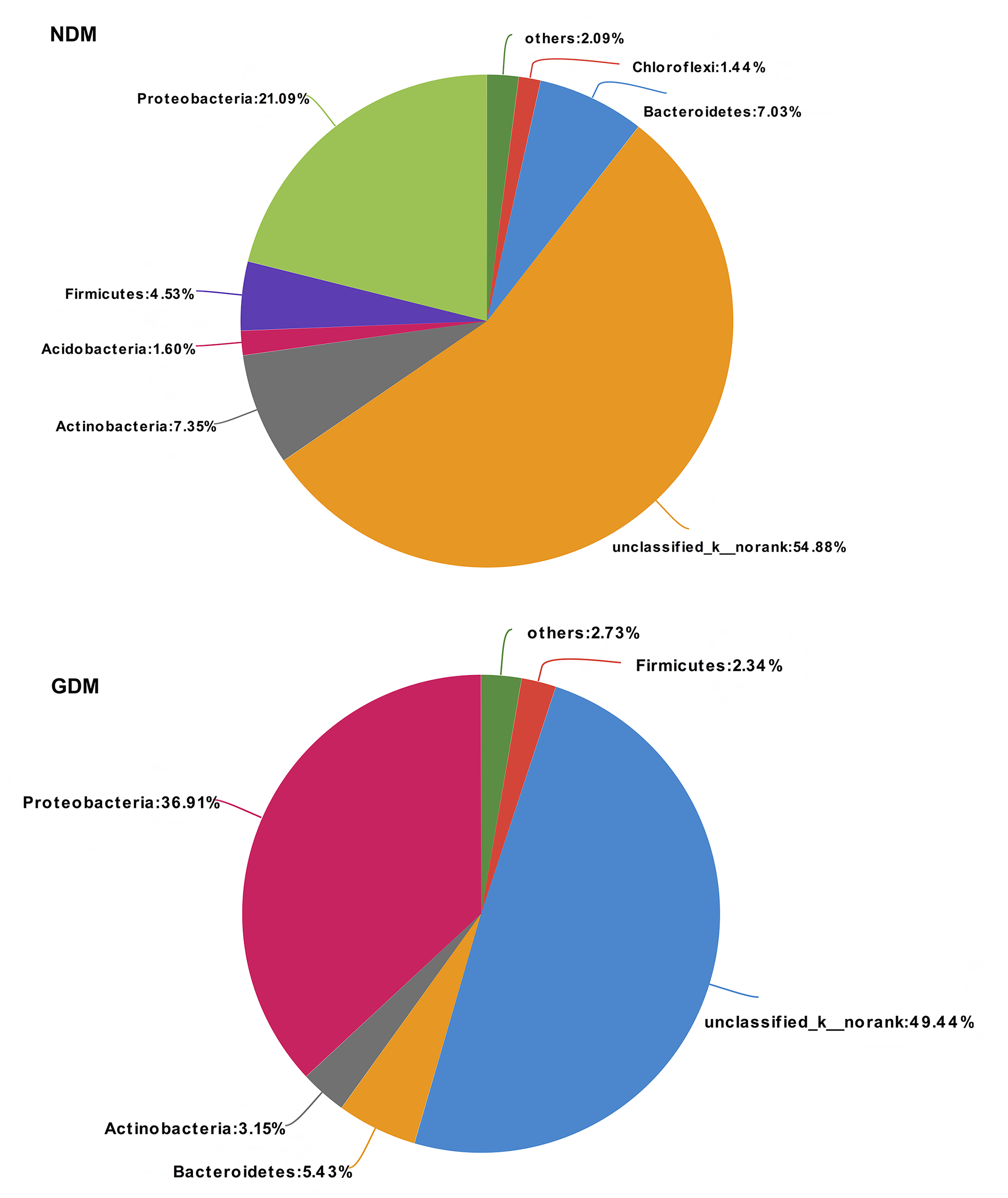

Supplement: Supplementary file 1 [file Image1.TIF]

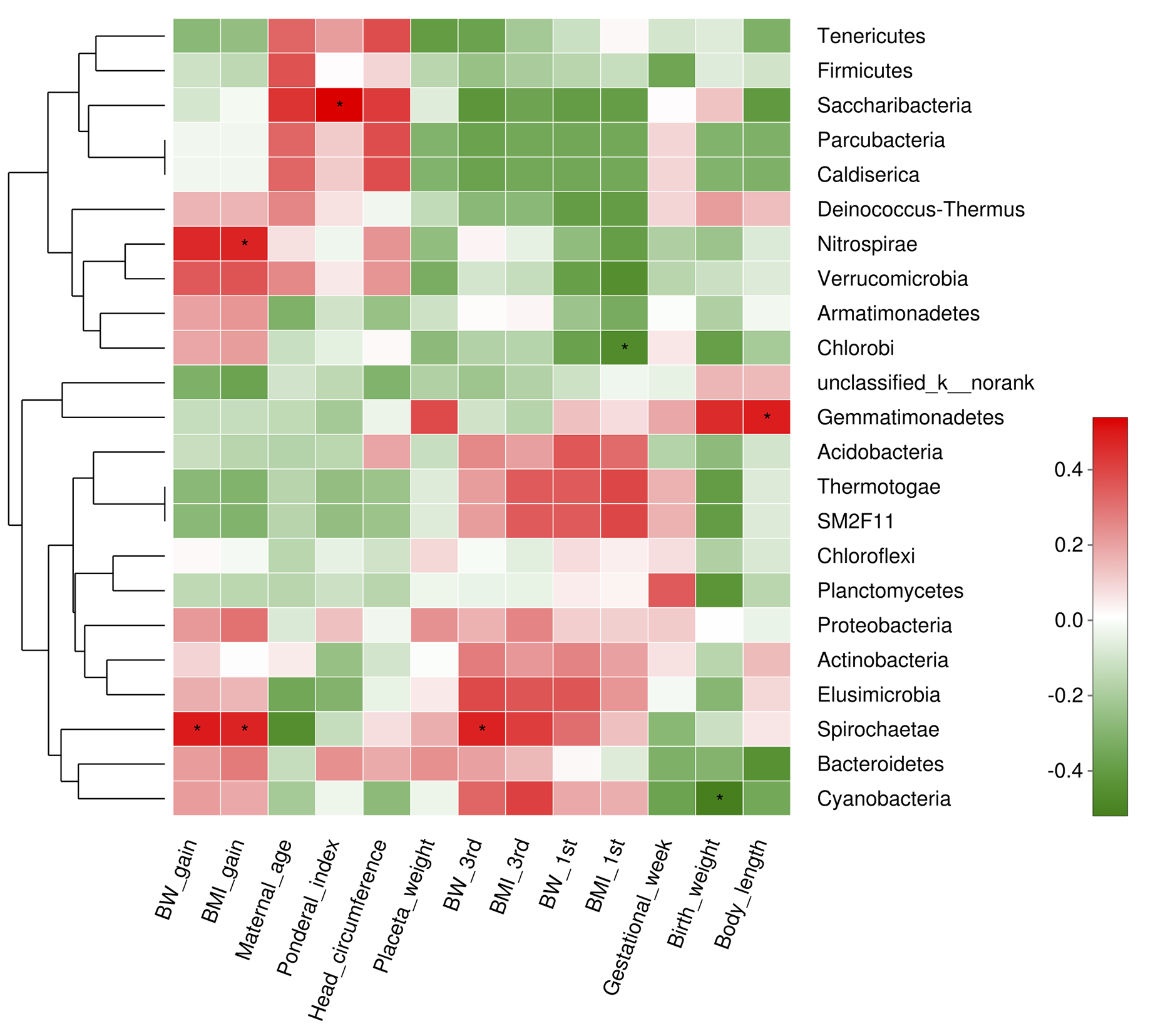

Supplement: Supplementary file 2 [file Image2.TIF]

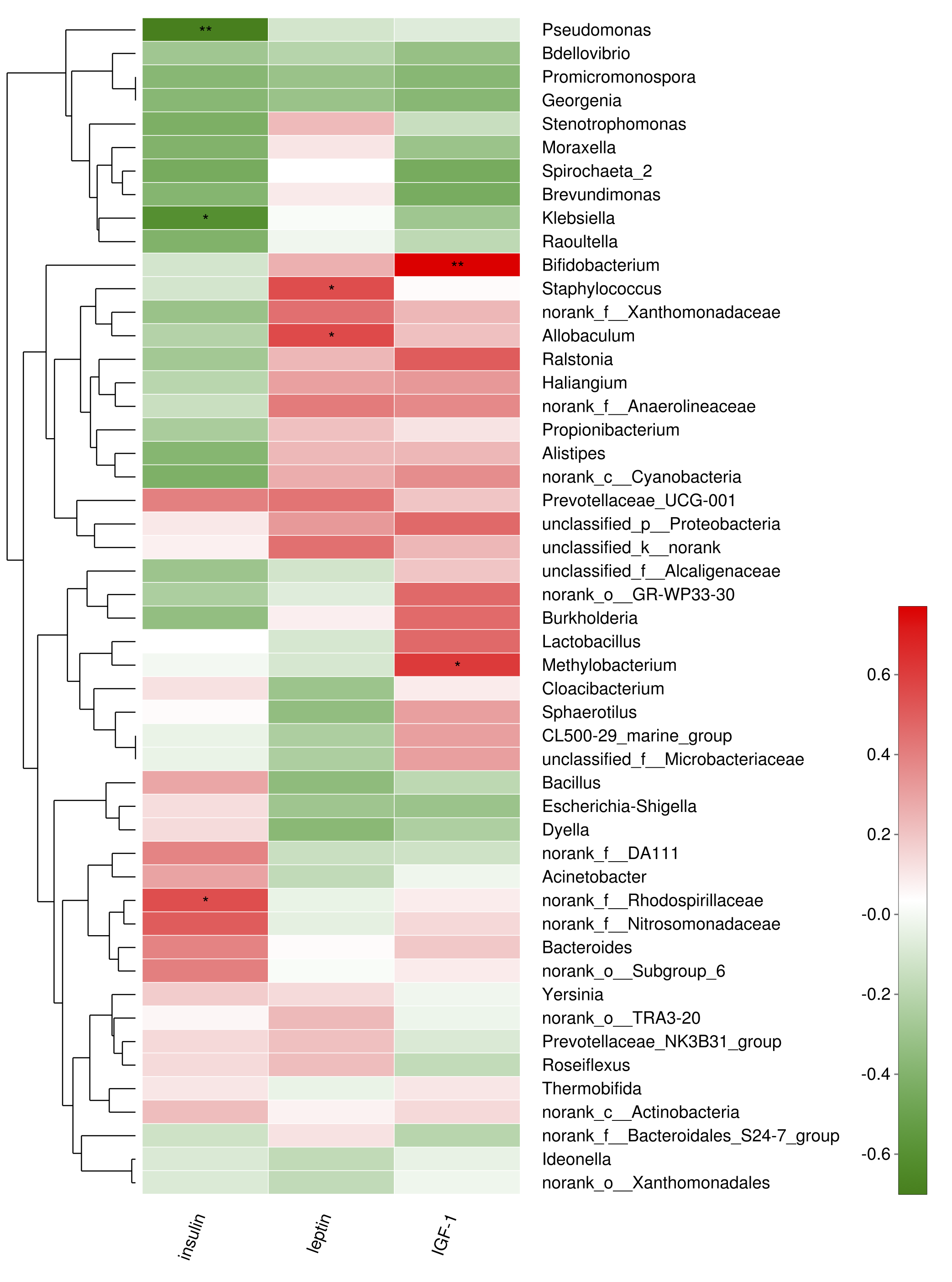

Supplement: Supplementary file 3 [file Image3.TIF]
