# Supplementary material for: The Placental Microbiota Is Altered among Subjects with Gestational Diabetes Mellitus: A Pilot Study
Source: Front Physiol. 2017 Sep 6;8:675. doi: 10.3389/fphys.2017.00675 (PMC5592210; doi:10.3389/fphys.2017.00675)
Supplement: Supplementary file 4 [file Table1.DOCX]

**Supplementary data**

**Figure Legends**

**Figure S1. Community analysis pie-plot shows the overall microbiota structure at the phylum level.** n = 10, in each group. GDM, gestational diabetes mellitus; NDM, Non-GDM.

**Figure S2. Correlation analyses between placental microbiota and clinical characteristics at the phylum level.** Correlation analyses were performed by using Spearman’s correlation analyses. n=10, in each group. (*) represents the specific genus whose abundance were significantly correlated with certain clinical parameters. The color of the spots in the right panel represents R-value of Spearman’s correlation between the genus and clinical parameters. BW, body weight; BMI, body mass index; BW 1st, body weight at the beginning of 1st trimester; BW 3rd, body weight at the end of 3rd trimester; BMI 1st, BMI at the beginning of 1st trimester; BMI 3rd, body mass index at the end of 3rd trimester;

**Figure S3. Correlation analyses between identified genera and cord blood parameters.** Correlation analyses were performed by using Spearman’s correlation analyses. (*) represents the specific genus whose abundance were significantly correlated with cord blood parameters. The color of the spots in the right panel represents R-value of Spearman’s correlation between genus and cord blood parameters. n=10, in each group. IGF-1, insulin-like growth factor-1.

**Table S1. Sequencing data and comparison of estimator indices between GDM and NDM groups.**

| Estimators | NDM | GDM | Pvalue |
| --- | --- | --- | --- |
| OUT | 176.25±27.86 | 159.50±34.57 | 0.283 |
| ace | 195.08±30.63 | 179.32±30.91 | 0.296 |
| chao | 197.59±30.78 | 184.69±34.85 | 0.424 |
| coverage | 99.9% | 99.9% | 0.870 |
| shannon | 3.47±0.51 | 3.11±0.77 | 0.274 |
| simpson | 0.10±0.08 | 0.14±0.13 | 0.496 |

Data represents as mean ± Standard Deviation (S.D.) Statistical analyses were performed with Mann‐Whitney U test between the two groups. The number of OTUs, richness estimator Chao, and diversity estimator Shannon were calculated at 3% distance. n=10, in each group. OUT, operational taxonomic unit.

**Table S2. The relative abundance (%) of placental microbiota differed significantly at the order level.**

| Taxonomic Rank | Specific Taxon | NDM (%) | GDM (%) | P value |
| --- | --- | --- | --- | --- |
| order | Burkholderiales | 4.977 | 13.280 | 0.046* |
| order | Bacteroidales | 6.043 | 2.445 | 0.023* |
| order | Rhizobiales | 0.207 | 1.287 | 0.011* |
| order | Erysipelotrichales | 0.393 | 0.000 | 0.047* |
| order | norank_c__Cyanobacteria | 0.278 | 0.000 | 0.006** |
| order | Holophagales | 0.029 | 0.000 | 0.017* |
| order | Hydrogenophilales | 0.020 | 0.000 | 0.017* |
| order | norank_p__Armatimonadetes | 0.007 | 0.000 | 0.017* |
| order | Brocadiales | 0.005 | 0.000 | 0.047* |
| order | Ignavibacteriales | 0.004 | 0.000 | 0.017* |

Data of macrosomia and control groups were showed as relative abundance (%) of phylum and genus in each group. Statistical analysis was performed by the Mann-Whitney U test. n=10, in each group. **P* < 0.05, ***P* < 0.001 GDM vs. NDM group.
